# Delayed mesh infection and mesh penetrating the transverse colon and small intestine after abdominal incisional hernia repair

**DOI:** 10.1093/jscr/rjaa409

**Published:** 2020-10-20

**Authors:** Kodai Tomioka, Toshihiro Fujioka, Tohru Satoh, Hidetoshi Makita, Rika Tsukui, Takeshi Aoki, Masahiko Murakami

**Affiliations:** Department of Surgery, Makita General Hospital, Tokyo, Japan; Division of Gastroenterological and General Surgery, Department of Surgery, Showa University, Tokyo, Japan; Department of Surgery, Makita General Hospital, Tokyo, Japan; Department of Surgery, Makita General Hospital, Tokyo, Japan; Department of Surgery, Makita General Hospital, Tokyo, Japan; Department of Surgery, Makita General Hospital, Tokyo, Japan; Department of Breast Surgical Oncology, Showa University Hospital, Tokyo, Japan; Division of Gastroenterological and General Surgery, Department of Surgery, Showa University, Tokyo, Japan; Division of Gastroenterological and General Surgery, Department of Surgery, Showa University, Tokyo, Japan

## Abstract

The occurrence of late-onset mesh infection and mesh invasion into the intestine after abdominal incisional hernia repair is extremely rare. Herein, we describe the first case of late-onset mesh infection and mesh penetrating the transverse colon and small intestine 5 years after incisional hernia repair using an expanded polytetrafluoroethylene mesh. The symptom was drainage from the reddish wound, and computed tomography scan revealed intestinal prolapse with local wall thickening. The mesh removal and small intestine and colon resection were conducted because the small intestine and transverse colon formed a mass containing the mesh inside. The events were caused by the lack of mesh fixation, and the dislodged mesh penetrating the intestinal tract caused the infection. For mesh infections in which conservative treatment is not effective, mesh removal and organ excision should not be delayed regardless whether there is a strong adhesion of the abdominal cavity.

## INTRODUCTION

Incisional hernia is an important complication of abdominal surgery. In recent years, the tension-free method has been mainly used since it has a lower recurrence rate than the conventional repair methods [1]. The incidence rates of early- and late-onset mesh infection after abdominal incisional hernia repair using a Composix mesh were ~0.9% and 0.4%, respectively [2], and the incidence rate of Composix mesh penetrating into the small intestine was ~1.1% [3]. The current study described a case of late-onset mesh infection and penetration into the transverse colon and small intestine 5 years after abdominal incisional hernia repair as the firstcase.

## CASE PRESENTATION

**Figure 1:**
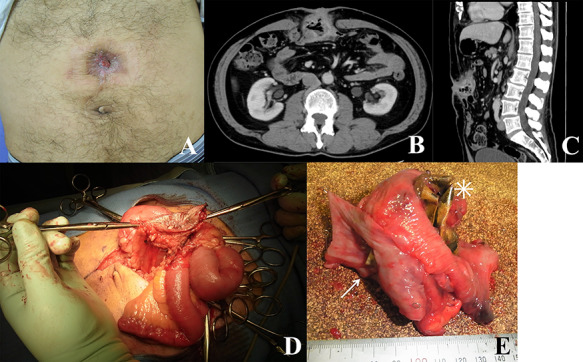
(**A**) Image of the patient’s abdomen before surgery. Reddish wound and fistula; (**B** and **C**) contrast-enhanced CT scan showing recurrence of abdominal incisional hernia with intestinal wall thickening; (**D**) intraoperative findings; skin, abdominal wall and intestines forming a mass; (**E**) excised specimen; mesh migrating through the small intestinal wall (asterisk) and its corner penetrating the colon (arrow).

A 61-year-old man underwent abdominal incisional hernia repair using an expanded polytetrafluoroethylene (ePTFE) mesh 5 years back. The patient was referred to our hospital due to drainage from the reddish wound in the abdomen (Fig. 1A). Based on the surgical record, the size of the wound along the previous surgical scar was 8 cm, and the mesh was placed in the abdominal cavity using the Ventrio mesh (Bard, Warwick, Rhode Island, USA) (8 × 12 cm) and was fixed with four stitches. Laboratory test revealed a slightly elevated C-reactive protein level at 0.83 mg/dL. The symptoms were believed to be caused by mesh infection, mesh removal surgery was planned to control infection. Contrast-enhanced abdominal computed tomography (CT) scan revealed recurrence of abdominal incisional hernia with local intestinal wall thickening (Fig. 1B and C). The surgery was performed by cutting along the midline of the operative scar approximately 15 cm, the intraoperative finding was high degree of adhesions in the abdominal cavity. Moreover, the small intestine and transverse colon formed a mass, and the mesh was not visible but palpable (Fig. 1D). Two parts of the small intestine and transverse colon resection were resected, and three anastomoses were created. In terms of the remaining rectus sheath and the site of infection, direct suturing was performed. Based on the examination of the excised specimen, most part of the mesh had penetrated and was exposed in the small intestine, the corners of the hardened and bent mesh penetrated the transverse colon and the other parts the small intestine (Fig. 1E). Postoperative wound infection was observed and it was treated with abscess drainage, the patient was discharged 24 days after the surgery. On histological examination, the mesh penetrated the small intestine and colon, and the small and large intestines were lumped together causing inflammatory fibrous adhesions.

## DISCUSSION

The treatment methods for abdominal incisional hernia include the simple closure method and tension-free method using a mesh, which depend on the diameter of the hernia orifice. The simple closure method is not suitable when the hernia orifice is ≥4 cm. The recurrence rate of this method is ~60%, and that of the mesh method is ~30%. Thus, the mesh method has been widely used recently [4]. The important complication of the tension-free method is mesh infection, and the condition is classified into early- and late-onset (6 months after repair) infection. Interestingly, the occurrence of late-onset mesh infection is rare, with only five cases recorded in the literature to date (Table 1). The causative agents differed, which are demonstrated in Table 1. In the current case, the bacteria identified were *Klebsiella pneumoniae*, *Escherichia coli* and *Enterococcus faecalis* [5–9]. In only one case, the ePTFE mesh was stuck in the jejunum [7]. The initial treatments are abscess drainage and antibiotic therapy. In the current case, wound drainage and antibiotic treatment did not improve the abscess, and surgical treatment was considered appropriate. CT scan revealed a strong adhesion of the intestinal tract, surgical treatment was not performed immediately as manipulation during surgery is challenging and this method could be overly invasive. However, the mesh was found to be crumpled and it penetrated the intestinal tract. Therefore, no matter how severe adhesion exists, conservative therapy is not sufficient in completely treating the mesh infection.

**Table 1:** Studies of late-onset mesh infection after abdominal incisional hernia repair published in the literature

Author	Year	Age	Sex	Country	Period after operation	Causative agent	Mesh penetrating
Bliziotis *et al.* [5]	2006	59	Female	Greece	6 months	*Staphylococcus aureus*	No
Jezupovs *et al.* [6]	2006	77	Male	Latvia	18 months	*Citrobacter koseri*	No
Foda *et al.* [7]	2009	50	Male	USA	36 months	*Escherichia coli*	Yes; Jejunum
Mohamed *et al.* [8]	2016	44	Female	USA	60 months	*Pseudomonas aeruginosa*	No
Tamura *et al.* [9]	2019	66	Male	Japan	120 months	*MRSA*	No
Present case	2020	61	Male	Japan	60 months	*Klebsiella pneumoniae, Escherichia coli, Enterococcus faecalis*	Yes; small intestine and colon

MRSA, methicillin-resistant *Staphylococcus aureus*.

Late-onset mesh infection was attributed to three mechanisms, which were as follows: (i) remnant infection due to primary surgery accompanied by contamination, (ii) bacterial translocation followed by septic events and (iii) *de novo* infection, caused by either mesh-intestine fistula formation or by transcutaneous [9]. However, in the study of Foda *et al*. [7], *E. coli* was detected in the mesh. In the current case, late-onset mesh infection might have occurred, which was preceded by the mesh penetrating the intestine [7]. Particularly in the current case, the mesh was crumpled, and it penetrated the intestinal tract. Moreover, its corner was stuck in the intestine. Based on this finding, mesh fixation in the previous hernia surgery was inappropriate, and deformation of the mesh might have been affected by the gap between the abdominal wall and the mesh and the distortion of the mesh during suturing. Due to the low incidence of wound infection, the rectus abdominis muscle posterior surface, anterior peritoneal cavity, or abdominal cavity is better for mesh indwelling site. In addition, intraperitoneal indwelling can shorten the operative and fixation time and can reduce the separation area [2]. Incomplete peritoneal repair, inadequate fixation, or inappropriate amount of implantation space may account for the mesh migrating into the intraabdominal viscera, occasionally followed by fistula formation or mechanical bowel obstruction [10]. Furthermore, the sharp edge of the prosthetic mesh can cause injury in the viscera serosal layer. In the current case, fixing the mesh in the abdominal wall with four stitches was not sufficient. Rather, it must be securely fixed all around. However, intraabdominal positioning of the ePTFE mesh may be safe even if it comes in contact with the viscera when used properly.

The occurrence of late-onset mesh infection is relatively rare. To the best of our knowledge, this is the first case of infected mesh simultaneously penetrating both the small intestine and colon. Mesh removal and organ resection should be considered in cases of mesh infection in which conservative therapy, such as abscess drainage and antibiotic treatment, is not effective.

## Conflict of interest statement

None declared.

## Funding

None.
